# How Can We Optimize the Value Assessment and Appraisal of Orphan Drugs for Reimbursement Purposes? A Qualitative Interview Study Across European Countries

**DOI:** 10.3389/fphar.2022.902150

**Published:** 2022-07-19

**Authors:** Alessandra Blonda, Yvonne Denier, Isabelle Huys, Pawel Kawalec, Steven Simoens

**Affiliations:** ^1^ Department of Pharmaceutical and Pharmacological Sciences, KU Leuven, Leuven, Belgium; ^2^ Department of Public Health and Primary Care, KU Leuven, Leuven, Belgium; ^3^ Institute of Public Health, Faculty of Health Sciences, Jagiellonian University Medical College, Krakow, Poland

**Keywords:** orphan medicinal product (OMP), rare disease (RD), health technology assessment (HTA), value assessment, reimbursement, appraisal (evaluation)

## Abstract

**Introduction:** The expansion of orphan drug treatment at increasing prices, together with uncertainties regarding their (cost-)effectiveness raises difficulties for decision-makers to assess these drugs for reimbursement. The present qualitative study aims to gain better insight into current value assessment and appraisal frameworks for orphan drugs, and provides guidance for improvement.

**Methods:** 22 European experts from 19 different countries were included in a qualitative survey, followed by in-depth semi-structured interviews. These experts were academics, members of reimbursement agencies or health authorities, or members of regulatory or health/social insurance institutions. Adopting a Grounded Theory approach, transcripts were analysed according to the QUAGOL method, supported by the qualitative data analysis software Nvivo.

**Results:** Although participants indicated several good practices (e.g., the involvement of patients and the presence of structure and consistency), several barriers (e.g., the lack of transparency) lead to questions regarding the efficiency of the overall reimbursement process. In addition, the study identified a number of “contextual” determinants (e.g., bias, perverse effects of the orphan drug legislation, and an inadequate consideration of the opportunity cost), which may undermine the legitimacy of orphan drug reimbursement decisions.

**Conclusion:** The present study provides guidance for decision-makers to improve the efficiency of orphan drug reimbursement. In particular, decision-makers can generate quick wins by limiting the impact of contextual determinants rather than improving the methods included in the HTA. When implemented into a framework that promotes “Accountability for Reasonableness” (A4R), this allows decision-makers to improve the legitimacy of reimbursement decisions concerning future orphan drugs.

## Introduction

Even in the 21st century, 95 percent of rare disease patients are still confronted with an unmet medical need, with their treatment options being largely inadequate. As orphan drugs - intended for the treatment of a rare disease that affects less than one in 2,000 people - are being developed and launched onto the market at increasing prices, their burden on national healthcare budgets increases (Gombocz and Vogler 2020). Meanwhile, due to their high price and/or often uncertain effectiveness, these drugs often exceed conventional incremental cost-effectiveness (ICER) thresholds (Drummond et al., 2007; Kanavos and Nicod 2012; Drummond and Towse 2014; Douglas et al., 2015; Chambers et al., 2020). As a result, decision-makers are increasingly adapting their standard reimbursement processes (usually consisting of a value- or health technology assessment (HTA), followed by an appraisal) in order to cater to the specific characteristics of orphan drugs and rare diseases. For instance, several jurisdictions have implemented separate pathways for orphan drugs or ultra-orphan drugs, allow a variable ICER threshold, or have implemented other methods to allow more flexibility during the value assessment and/or appraisal process of orphan drugs or rare diseases (Nicod et al., 2020a; Ollendorf et al., 2018; Nicod and Whittal 2020). This variety of approaches risks to contribute to unequal access by patients to these drugs throughout the EU (Stawowczyk et al., 2019; Zamora et al., 2019; Czech et al., 2020).

Meanwhile, despite the majority of countries adapting their standard reimbursement processes, orphan drug prices - and by extension value assessment frameworks (VAFs) and appraisal processes - have been subject to a lot of scrutiny (Simoens 2011; Hughes-Wilson et al., 2012; Picavet et al., 2014a; Luzzatto et al., 2018). In part, this criticism is rooted in the secrecy surrounding the prices and conditions that are often negotiated under the terms of an increasing number of managed entry agreements (MEAs). For orphan drugs for which there is still substantial uncertainty regarding their cost- and/or effectiveness, a MEA allows a temporary reimbursement on provision of a price reduction (financial-based MEAs) or on condition that additional data on real-world effectiveness are collected in a dedicated disease or treatment registry (outcome- or performance-based MEAs). However, due to the lack of transparency surrounding their content, it is often unclear whether the orphan drug meets the conditions as defined in the MEA.

Previous research has focused on VAFs and appraisal processes for reimbursement purposes of (ultra-) orphan drugs in general (Nicod et al., 2020b; Annemans et al., 2017; Nicod et al., 2019; Sussex et al., 2013a; Wagner et al., 2016; Schey et al., 2017) or of particular orphan drugs (Abdallah et al., 2021; Whittal et al., 2021; Blonda et al., 2022). However, there is still a shortfall of actionable and practical guidance directed at decision-makers who wish to improve their VAF and appraisal process for orphan drugs, as a means to optimise the reimbursement process as a whole and attain more substantiated outcomes. Our previous study focused on the strengths and barriers related to VAFs for orphan drugs from a theoretical perspective (Blonda et al., 2021). The aim of the present, qualitative study is to investigate how orphan drug reimbursement experts perceive the VAF and appraisal process for orphan drugs in their country. Building on this experience and viewpoints, we provide specific and practical recommendations for decision-makers on how to improve their current reimbursement processes for orphan drugs.

## Methods

### Design

A Grounded Theory approach was applied to analyse the viewpoints of interviewees concerning the value assessment and appraisal frameworks for orphan drugs in their respective jurisdictions. Grounded theory is an inductive approach which allows to derive theories from the data, and the qualitative research design makes it a suitable approach for uncovering the underlying factors that may influence the value assessment of orphan drugs, as shared by the interviewees (Corbin and Strauss 2015).

### Sample

Participants were included if they had experience with or knowledge about the assessment and appraisal process of orphan drugs in at least one European country or jurisdiction. Initially, participants were recruited via a contact person from the European Piperska Consortium. This consortium wishes to ensure that Europe has robust systems in place in order to promote the rational use of drugs - among which orphan drugs - as a means to improve health. Its members are mostly professors, researchers and/or health economists who work for national or regional health services or sickness funds, where they are involved in the reimbursement, formulary listing and/or enhancing the rational medicine use. They are regarded as experts in their field, and one of the co-authors (SS), is one of the Consortium’s members. The Piperska contact person extended the study’s invitation to the members, wherein they were asked to reply if they met the inclusion criteria. In addition, potential candidates were identified via the networks of the members of the research team, via author lists of peer-reviewed arcticles on the topic of market access to orphan drugs, via websites of institutions concerned with health technology assessment (HTA) or reimbursement of orphan drugs, or via the social networking website *LinkedIn*. We applied purposive sampling with the intention to recruit a participant group that was geographically spread throughout the EU. The aim was to recruit at minimum one participant per country.

In total, we recruited 22 participants, 12 male and 10 female experts, from 19 different countries to participate in a qualitative survey and semi-structured in-depth interview (Table 1). The majority of the participants were senior-level (*n* = 14) with a hetegorenous professional background, mainly active in a reimbursement or HTA agency (*n* = 8), in academia (*n* = 7), or at a health or social insurance fund (*n* = 6).

**TABLE 1:** Characteristics of participants.

Characteristics of participants (*n* = 22)	
Gender	
Male	12
Female	10
Experience level	
Junior (<5 years experience)	1
Mid-level (≥6 years experience)	7
Senior (≥15 years experience)	14
Professional occupation^a^	
Academic	7
Reimbursement or HTA agency	8
Consultancy	3
Regulatory or policy making institution	4
Health or social insurance institution	6
Industry	1
Country of origin (*n* = 19)	Belgium, Wales, Lithuania, Slovenia, Bulgaria, Estonia, Ireland, Italy, Croatia, Malta, Austria, Poland, Romania, Scotland, Slovakia, Spain, Czech Republic, Sweden, Bosnia and Herzegovina

a Per participant, combinations of occupations can apply. Abbreviations: HTA, health technology assessment.

### Data Collection

We collected data via a qualitative survey and via one-on-one, semi-structured in-depth interviews, which were completed between the 25th of August 2020 and the 23rd of March 2021.

We prepared a preliminary interview guide based on a review of international, peer-reviewed literature, legal and policy texts on the value assessment or appraisal process of orphan drugs. We asked the participants to fill in a qualitative survey, which was based on a standard reporting template and consisted of open- and closed-ended questions. The questions in the survey inquired into the characteristics of the reimbursement process in the participant’s respective country and, more specifically, about combinations of approaches towards the value assessment and/or appraisal process of orphan drugs. Subsequently, we used the survey results to refine the semi-structured interview guide for each interviewee individually, which allowed a deeper exploration of country-specific aspects during the interviews. The final interview guide consisted of open-ended questions and was used in a flexible manner, asking interviewees about the strengths and weaknesses of either implemented or hypothetical frameworks for orphan drugs and gathering proposals for the improvement of these frameworks. All interviews were conducted and recorded via Microsoft Teams, Skype or Zoom. The interviewer (AB) aimed for timeslots of 90 min per interview, depending on the availability of the participant. In three cases, AB scheduled a follow-up interview due to time restraints of the first interview session. The majority of the interviews were conducted in English (*n* = 20), two interviews were conducted in Dutch. At the end of each interview, participants were asked whether there were any themes that were not covered yet. Ultimately, data were only included from participants that participated both in the survey and the interview.

### Ethical Considerations

The research protocol was approved by the Ethical Committee (EC) Research University Hospitals Leuven (6/05/2020, S63958). Each participant signed the informed consent prior to filling in the survey. We ensured the anonymity of the participants, unless otherwise discussed and agreed with the participant. During and after the study, all data were treated confidentially.

### Data Analysis

All interviews were conducted by the same researcher (AB) and transcribed *ad verbatim* (including non-verbal signs) by working students affiliated with KU Leuven. The Qualitative Analysis Guide of Leuven (QUAGOL) was followed to guide the process of qualitative data analysis (Dierckx de Casterle et al., 2012). In a first stage, researcher (AB) performed a first “pen and paper” analysis of a small amount of interviews, highlighting key concepts and preparing a conceptual scheme or mindmap for each interview. During an iterative process of analysis and discussion, AB and one of the supervisors (YD) scheduled periodic calls in which main concepts and schemes of an increasing amount of interviews were identified and discussed. Concomitantly YD read the interviews and applied a fitting test to ensure a match with the mindmaps, in order to guarantee the validity of the analysis and minimize bias.

The iterative discussions allowed to transition from interview-specific concepts to an overarching structure that presented the main key concepts that recurred throughout the interviews. After performing the first-stage analysis for one third of the interviews, a meeting was planned between AB, YD and SS, in which both SS and YD read two of the richest transcripts and their mindmaps, as well as two other mindmaps (without transcripts). This allowed the research team to further discuss, structure and refine the key concepts identified so far. After finalizing the first-stage analysis of approximately 50 percent of the interviews, AB initiated the second stage of the analysis by digitally coding the interviews in *NVivo 1.5*, a qualitative data analysis software. After finalizing the first and second-stage analysis of the interviews in a consecutive manner, an overarching peer review and debriefing took place (October 2021) with all members of the research team (*n* = 7). During this plenary review and debriefing meeting, the interpretation of the transcripts was validated by means of an in-depth discussion of the overarching structure in which the key concepts were presented. In preparation of this plenary meeting, each member of the research team read two transcripts and their matching mindmaps. Ultimately, this plenary meeting validated the accuracy of the overarching structure of key concepts in comparison to the transcripts. From the transcripts, quotes were selected that best complement or illustrate the concepts described in the results section. Deleted parts of the quotes are indicated by a “[…]” and additional information is provided between square brackets. The coding [P1, P2, …] is linked to the participant.

Following transcript analysis, AB qualitatively identified several criteria that may be considered to signify a “good practice” when setting up a framework for the value assessment and appraisal of orphan drugs. Afterwards, AB examined the performance of the participating jurisdictions or countries against these criteria on an aggregated level. For each good practice, AB inductively explored whether these were predominantly present (green), whether the current practice could be improved (orange) or whether the good practice was overall absent (red).

## Results

The following sections will discuss the elements that influence access to orphan drugs and the specific reimbursement processes that are applied to provide access *(Sections Elements That Influence Access to Orphan Drugs* and *Specific Reimbursement Processes to Provide Access to Orphan Drugs*). Subsequently, this study will describe the positive attributes, barriers (*Section Factors Impacting the Reimbursement Process of Orphan Drugs*) and external factors (*Section External Factors Influencing the Appraisal of Orphan Drugs*) that influence the value assessment and/or appraisal process. As a way forward, interviewees presented several concrete steps for decision-makers to improve their value assessment and appraisal framework for orphan drugs. In addition, the analysis generated a list of five criteria that participants considered to signify a good practice when setting up or adapting the reimbursement framework for orphan drugs (*Improving the Value Assessment and Appraisal of Orphan Drugs: Ways Forward as Proposed by Participants*).

### Elements That Influence Access to Orphan Drugs

Overall, the results highlighted several elements that may impact whether a country factually provides access to an orphan drugs. These variations are caused by various factors, such as the country’s financial resources, which are reflected in its gross domestic product (GDP) and consequently also in its healthcare budget.

In addition, the presence of an international reference pricing system (IRP) may influence the reimbursement process as it largely determines time-to-access.

*“Companies have a free choice to pick the countries which they like, which they prefer. And it happens that less affluent countries just don*’*t get these drugs. Because the companies are not interested to come there. Because of lower price or, you know... But this is the weakness on EU level, which has been addressed many times. Yet the Commission doesn’t believe that they can force companies to come to smaller, less attractive markets.”[P12]*

At policy-level, the access to orphan drugs is dependent on whether or not rare diseases are considered to be a priority and are defined as such in national legislation.

*“The unmet need is there, the need to treat. Yet no legislation defines that the rare disease patient has to be treated. If they [the authorities] say that they don’t want to treat him because they can’t afford it, it is acceptable for everybody in this country. Okay you can go for justice, but this lasts many years and even if you win, you will lose your patient or your family member.”[P5]*

Finally, a country’s culture and value framework concerning disease burden, public health, healthcare services and priorities define how orphan drugs are assessed. A country’s culture, for instance, may influence the interpretation of disease burden as assessed by the EQ-5D, a standardized questionnaire that allows to define and subsequently value health states.

*“You have to have at least a scale of 10 in order to capture all the tiny improvements, all the nuances. You can not say for sure… And there are cultural things. In some cultures it is better to be dead than to suffer, to be in bed, immobilized with a depression, depending on somebody, totally immobilized in pain. So it is hard to say that this kind of measurement would bring any added value.”[P13]*

### Specific Reimbursement Processes to Provide Access to Orphan Drugs

In general, participants confirmed that countries apply various reimbursement processes in order to provide access to (ultra-) orphan drugs.

First of all, most countries are either implicitly or explicitly adjusting their standard VAF to the specifics of (ultra-) orphan drugs or drugs intended for rare diseases. In particular, several participants indicated that their frameworks allow more flexibility such as a higher ICER threshold or accepting different quality of clinical data. In addition, many jurisdictions include ethical considerations such as equal access to health, exemplified by the assessment of reimbursement criteria such as unmet need or disease severity. A minority of the countries have a separate framework for ultra- orphan drugs. They are granted access to these frameworks depending on disease prevalence, or depending on whether the ultra-orphan drug meets a combination of specific requirements, which consider patients’ unmet needs, the drug’s additional health benefits and others. In some cases, these separate frameworks also include a hearing dedicated to patients, during which they can share their experiences. Other variations depend on the extent to which the decision-making process is structured. For instance, there exist variations in the way in which criteria are scored (either quantitative or qualitative). Disparities also exist in whether criteria are considered in an implicit rather than explicit manner. Moreover, several countries have attempted to structure the assessment process in a decision-matrix, resembling a multi-criteria decision analysis (MCDA). In a MCDA, a multi-stakeholder team may define and/or weigh reimbursement criteria, and afterwards scores the performance of the drug against these criteria by means of a MCDA matrix. This matrix, which is published afterwards, includes the arguments or scores of all stakeholders related to each criterion and the subsequent reimbursement decision. For (ultra-) orphan drugs, these jurisdictions may allow a more lenient point system for ultra- orphan drugs as opposed to orphan drugs and non- orphan drugs within the context of these matrices or MCDA.

Second, several countries provide a temporary reimbursement for orphan drugs or ultra- orphan drugs (usually for a period of 3 years) under the conditions of a MEA. In the case of a financial-based MEA these may, for instance, consist of a simple discount on the list price (sometimes up to 90%) or a cost-volume agreement (where discounts are linked to the number of treated patients). In the context of an outcome-based agreement, reimbursement may be limited to responders only.

Third, in addition to their national reimbursement process, the majority of countries provide reimbursement on a patient-named basis. This reimbursement pathway is intended for one patient in particular and is applied more regularly in the context of (ultra-) orphan drugs as a result of disease rarity. Usually, the process is initiated by the clinician and is approved and financed by an insurance fund. In general, an orphan drug reimbursed on a patient-named basis does not involve a complex submission dossier. Yet, the reimbursement decision is mostly based on the clinician’s judgement. Finally, some countries have created separate funds in order to pay for orphan drugs.

### Factors Impacting the Reimbursement Process of Orphan Drugs

Table 2 summarizes the main aspects that experts perceive either as positive attributes or as barriers related to reimbursement processess for orphan drugs. A detailed discussion is provided below.

**TABLE 2:** Positive attributes and barriers perceived by experts, concerning reimbursement processes for orphan drugs.

Positive attributes	Barriers
Cooperation and communication between authorities and the industry	Lack of data
Transparency	Lack of expertise
Presence and inclusion of ethical arguments	Fixating the reimbursement criteria/framework
Involvement of a multi-stakeholder team, including patients	Involving the right stakeholders
Structure and consistency	Lack of trust
	Imbalance in negotiation power
	Lack of transparency
	Questions concerning cost-effectiveness of the overall reimbursement process

#### Positive Attributes Ascribed to Specific Applied and Preferred Reimbursement Processes for Orphan Drugs

##### Cooperation and Communication Between Authorities and the Industry

First of all, several participants indicated that cooperation and communication with the industry proved to be key for an efficient reimbursement of orphan drugs. Examples of good practices included early dialogue, in-process meetings, being informed about the (ultra-)orphan drugs in the development pipeline of the industry, for example through the practice of horizon scanning.

*“The more we can do to support companies and let them know what we are looking for, the more successful we will be.”[P4]*

##### Transparency

Also, although the majority of the participants appreciated the overall transparency that is present at the level of the reimbursement criteria, there is significant room for improvement on the level of the discussions - usually held behind closed doors - throughout the HTA/value assessment and appraisal process and in the reimbursement report that is published afterwards.

*“I think if you are clear and you are transparent on your decisions and the reason why you are moving (a threshold) up or down, everything is valid, no?”[P2]*

This is because transparency on the arguments behind a reimbursement decision informs and educates stakeholders such as patients, the public and the industry on the choices in healthcare spending and creates understanding of a reimbursement decision.

*“It comes back to the public understanding of what we do and educating them better, and that is quite hard work. […] but I think it becomes very difficult when you are the patient or the mother of the child with the condition, because your logic goes out the window regardless of what decision is made. I think being as clear as to why you made the decision you have made is important.”[P4]*

As these choices are even more prominent in the context of orphan drugs, where decision-makers feel more pressure to grant reimbursement, increased transparency would enable them to justify a negative reimbursement decision.

*“It is easier for them to say okay look, we have all the information on the table, we have acquired an elaborate system that looks into all evidence and if the evidence does not speak in favor of reimbursing that treatment then well that’s a fact, I mean I can’t change the facts.” [P1]*

In addition, transparently sharing the arguments in favor or against reimbursement may serve as a reference for future decisions and, hence, increase consistency in decision-making.

##### Presence and Inclusion of Ethical Arguments

In addition, most participants expressed the importance for an orphan drug reimbursement process to balance reimbursement criteria that reflect efficiency in spending, such as *cost-effectiveness*, against reimbursement criteria that respect the principle of solidarity and equity. This is due to the fact that rare disease patients are considered to be more unfortunate compared to non-rare disease patients, since generally, they suffer from mostly severe diseases of which little information is available, which results in an unmet medical need. For this reason, participants overall found *unmet medical need* and *disease severity* (either considered implicitly or explicitly) to be highly valuable reimbursement criteria. However, they underlined the need for a clear definition of the concept *unmet medical need*. Meanwhile, although several participants mentioned other reimbursement criteria such as *disease rarity*, *fairness*, a *patient’s group young age* and the *unavoidability* of providing treatment, the appropriateness of including these criteria in the reimbursement process showed to be inconsistent throughout the interviews.

*“It doesn’t matter if you die from spinal muscular atrophy or breast cancer: you are just as dead, you are not any more dead because you die from spinal muscular atrophy than if you die from prostate cancer.”[P13]*.

Though overall, participants did not find *disease rarity* to be an appropriate reimbursement criterium, several acknowledged its significance as a major driver of orphan drug prices.

*“I think if the cost of developing these gene therapies is a million pounds per patient and it is not a case of that being all the profit margin then there is debate to be had. And there is a room to accept and understand that within a developed health care system there could be a role for that.”[P11]*

##### Involvement of a Multi-Stakeholder Team, Including Patients

The majority of the participants also highlighted the importance of involving multiple stakeholders during several stages of the decision-making process. A multi-stakeholder group enriches the discusson leading up to the decision-making, as it aids to interpret the evidence from different viewpoints. In particular, the involvement of patients was considered an asset for most participants (on the condition that they are well informed), especially when they serve as advisors rather than decision-making entitees. Nevertheless, many participants had reservations, such as the difficulty in selecting the right “type” of patient (either suffering from the respective rare disease or not), potential ties of the patient or the patient organisation to the industry and a patient’s inherent bias in wanting their treatment to be reimbursed. A minority of jurisdictions organise separate hearings, which participants considered to be instrumental for including the patient voice without granting them decision-making power. However, such patient hearings may also introduce bias into the process, as we will discuss later.

##### Structure and Consistency

Most stakeholders preferred the presence of a certain degree of structure and consistency (in the value assessment and appraisal) instead of undefined and unclear rules, which they believe leads to case-by-case treatment and inconsistent decision-making. Such a structure may include benchmarks such as an ICER threshold or weighted quality-adjusted life year (QALY) measurement and leaves room for a flexible approach while maintaining a high level of consistency.

*“I think it is a good idea to try to quantify, to classify. But I think it is also a good idea to accept the fact that you will never actually be finished. […] It will always have to be continuously updated and revised and changed and worked on.” [P13]*

Nevertheless, several experts stressed the fact that the appraisal process should remain a humane, multidisciplinary judgement, particularly in the context of ultra-orphan drug appraisal.

*“When it comes to ultra-orphans, having a flexible, pragmatic process means that there is a lack of clarity. Particularly for companies when they are putting their submissions in. And also I guess when it comes to the public and understanding the decisions. […] If you want things to be really clear and you want all these factors in your process and weighted quite clearly, then you lose your pragmatism and it just becomes a formula and you don’t actually need a committee to make the decision. It is it is no longer a judgement. I think all the decisions we make are judgements and you need that flexibility.”[P4]*

An illustration of a reimbursement framework that presents a high level of structure and consistency is multi-criteria decision analysis (MCDA). Participants’ viewpoints regarding this specific framework are summarized in Box 1.

BOX 1 **The impact of MCDA on the value assessment framework for orphan drugs**

One particular and well-known tool to structure the value assessment and appraisal process is MCDA, for which participants shared their viewpoints from either a hypothetical or practical viewpoint. Participants who were not familiar with the practice focused mainly on their reservations towards its adoption: they feared that the framework would be too prescriptive by nature, too complex and too time-consuming, stressing the lack of human resources to successfully implement and conduct a MCDA for an (ultra-)orphan drug. Indeed, participants from jurisdictions that apply some form of MCDA confirmed that the framework may look well on paper but in practice still relies on the available resources and national priorities concerning access to OMPs. Meanwhile, they found that the framework structures and standardizes the process, allowing the criteria to be discussed step by step in a deliberative process. In addition, the overall reimbursement process and outcome may become much simpler for politicians to understand when the assessment and appraisal process are linked to a simple score system.

#### Barriers Related to Specific Applied and Preferred Reimbursement Processes for Orphan Drugs

##### Lack of Data

First of all, participants highlighted that the value assessment of orphan drugs is often hampered by a lack of data, for instance, due to insufficient evidence on their effectiveness and a lack of local data on cost and utilities.

*“Very few patients, very short time frames, usually the other thing is clinical endpoints that probably are difficult to relate to clinical practice. There is a whole lot of things that are difficult with this whole area.”[P6]*

In particular, several interviewees indicated the challenges related to the high costs associated with validating the EQ-5D in their country. A paucity of data may eventually inhibit a proper economic evaluation and creates barriers for setting up outcome-based MEAs, which are more complex (than pure financial-based MEAs) as they may link reimbursement to patients achieving (surrogate) endpoints.

“*Sometimes the cost-effectiveness is hard to calculate because of the lack of data on efficacy and safety. And well the drug ends up not being reimbursed for that.”[P8]*

Furthermore, absence of local data on disease prevalence complicates budget impact estimations and creates challenges when defining whether these drugs may access an adjusted or separate reimbursement pathway, especially in the case of an ultra-rare disease.

##### Lack of Expertise

Many participants also highlighted that the lack of national expertise is a particular problem in the context of rare diseases and orphan drugs, either to assess the data and analyses that were submitted, to perform the economic evaluation or to negotiate successfully.

*“In order to be a good negotiator you have to be very well trained and know everything, or almost everything. You should know how it works better, or at least as good as the partners. And in countries where this happened, they were able to get the product for a good price.”[P3]*

##### Fixating the Reimbursement Criteria/Framework

In addition, participants indicated that the lack of a structured reimbursement framework (for instance without fixed criteria and/or benchmarks) often complicates the negotiation leading up to the appraisal of the orphan drug.

*“You are stepping in the dark and just trying, so you are losing time, you are losing resources. If there are some criteria, whatever the criteria are, it could be much easier as a starting point at least. […] If you are going from the stretch and on case-by-case basis it is really frustrating and there is not any kind of rule. And there is no transparency in the end, because […] you don’t know what the conditions of the reimbursement are so you cannot benchmark, or see what is actually behind this decision”.[P10]*

Meanwhile, many participants acknowledged the difficulty in fixating criteria or assigning weights. First of all, they fear that an approach that is too rigid would turn the appraisal into an automatic rather than a reasonable and balancing process with a humane and patient-based perspective. Second, decision-makers struggle to balance “hard” and quantifiable criteria (such as *cost-effectiveness*) with “soft” criteria that are difficult to quantify (such as *severity* or *unmet need*).

*“I think it is difficult for a committee member to take this quite robust clinical evidence that is all nicely numbered and quantitative, and then this piece of softer patient-based narrative. I think it is quite difficult to bring those together. So if there is a way that we can put more robustness into the patient-based evidence I think that will help. I don’t have the answer to that by the way.”[P4]*

##### Involving the Right Stakeholders

Furthermore, the lack of expertise in the area of rare diseases leads to barriers regarding the involvement of multiple stakeholders in the decision-making process.

“*Finding clinicians to be involved is also quite difficult because usually the clinicians who are the specialists in a certain area, especially when it comes to orphans or ultra-orphans, are also heavily involved in clinical trials with the companies.*”*[P13]*

This may compromise the neutrality of stakeholders and may contribute to a lack of trust between the members of the reimbursement committee.

##### Lack of Trust

In fact, the lack of trust between stakeholders was shown to be a major barrier on several levels of the reimbursement process. For instance, it prevents health insurance funds or other health authorities from sharing aggregated data with the industry (for instance on rare disease prevalence) which in turn may lead to a distrust of the (prevalence) data that was submitted by the pharmaceutical company. Overall, the lack of trust keeps parties from communicating effectively and consequently hampers an efficient value assessment, which is ideally based on robust and trustworthy data.

*“They think it is good that we don’t meet the industry because the industry will influence us. Which is acceptable from one point of view, but from the other point of view it is a problem, because they can explain many things for which you don’t have time to study […] because you have limited capacity and so on.”[P5]*

##### Imbalance in Negotiation Power

The combination of the lack of clear structure or criteria, the paucity of national expertise and a general distrust between the stakeholders leads to power imbalances in negotiation, in favor of the industry.

“*I know that in business you may ask more just to get less. But it is difficult to do that in countries where the power of negotiation from the authorities is not so developed or the legislation is not allowing to have negotiations of any kind.*”*[P3]*

These imbalances are further amplified by the fact that not all stakeholders are equally or sufficiently prepared or informed.

*“Those people don’t look at their representation in the committee as a job. Because they are not reimbursed for their time. That is a solid reason I think, but they come unprepared and that is why I have to re-present my assessment because it is the first time they hear things.”[P18]*

It also has implications regarding the nature of the voting process as the voice of the “loudest” often implicitly carries the most weight. For instance, several interviewees pointed out that in many cases the Government has a disproportionately large role in the final vote, which in part could be attributed to their vigourous preparation.

##### Lack of Transparency

In part, these barriers result from a lack of transparency while at the same time contributing to its continuation on several levels: from the lack of transparency on the reimbursement criteria and their weighing to the timelines of the overall reimbursement process. For example, the HTA or reimbursement report often does not reflect the discussion that took place between the value assessment and the appraisal or final decision. However, the tax payer has a right to access comprehensive information on the allocation of healthcare resources. Also, participants often feel that the true motives of the stakeholders involved during the decision-making remain hidden and, as a result, they may perceive the decision-making process as a game of politics.

*“It is only the play of the industry you know. Society has taken this play the industry has given them. And we, the health care institutions or the payers, are dancing on the music. But this is the music of the industry, just to hide the realistic prices, which are of course significantly lower. And then we play this pharmaco-economic play on the music. Yet this is something that we are lying about to each other.”[P5]*

Ultimately, the real drug prices are (usually) kept secret, due to concerns over parallel import, among other things.

*“Finding out what it actually costs to develop a new drug, I would rather try to dig my way through the Mount Everest with a spoon […] Of course they have the numbers internally but they will not tell you. The most closely guarded secret on this planet is how much it costs to develop a new drug.”[P13]*

##### Questions Concerning Cost-Effectiveness of the Overall Reimbursement Process

Finally, participants questioned the overall cost-effectiveness of the reimbursement process. While most orphan drugs do not meet the current cost-effectiveness thresholds, especially in countries with a low GDP, the decision-maker will often conclude that rare disease patients require access to their treatment regardless of its cost-effectiveness.

*“I understand that not every country can afford the treatment, but the question should be “how can we afford the treatment” […]? And not if the rare disease treatment is cost-effective because it will never be […] But on the other hand if we left it (the cost-effectiveness analysis) out then we are somehow okay with the fact that orphan-drugs will automatically not be cost-effective.”[P5]*

Consequently, the economic evaluation may seem like a lost measure in terms of time, resources and therefore, money.

*“And I remember that I read – I think one or 3 years ago, well some time ago – an article about: How can we affect the cost-effectiveness of the cost-effectiveness-analysis? […] if I would plan to get a Phd now, I would study the cost-effectiveness of the cost-effectiveness-analysis.”[P3]*

### External Factors Influencing the Appraisal of Orphan Drugs

#### Contextual Determinants as an External Factor Influencing Orphan Drug Appraisal

Although the barriers presented in Table 2 hamper an expedient value assessment and appraisal process, there are other factors that may have a negative impact on the appraisal/final decision. These factors seem to be dependent on the political, cultural or historical context of the appraisal process, which is why we will refer to them as contextual determinants hereafter. Throughout the interviews, we identified three main categories of contextual determinants as detailed in the paragraphs below.

##### Conflict of Interest and Bias

Participants often pointed out a risk of bias on several levels during the decision-making process. First of all, on the level of the committee, bias may arise when the stakeholders or clinicians involved simultaneously liaise with authorities, the insurance funds and the pharmaceutical companies. In combination with the lack of national expertise, this may cause a conflict of interest.

*“It starts with this prevalence, which is so complicated to get in a small country where you have one specialist, maybe one clinic for the disease, and this clinic should serve the industry, but they should also serve the Ministry of Health and the payer […]. So you are always somehow biased, and not really independent.”[P5]*

Second, bias may emerge when only patients with the disease are involved or consulted during the reimbursement process. This is mainly due to the perception that these patients are influenced by the industry, for instance through their patient organisation. Also, several participants pointed out that patients with the disease would generally be in favor of reimbursing any drug that may treat them. This becomes apparent especially in countries or jurisdictions where separate hearings are organised that enable patients to share their experience.

*“This meeting is only with a couple of patient groups and a patient who has received the drug is there […] So obviously the patients that died after receiving the drug are not at the meeting (laughs). I should not laugh at this but you see what I mean. So you are obviously getting quite a biased view from that patient [who is present at the meeting].”[P4]*

Third, participants highlighted that external pressure may also cause bias, for instance by politicians who are concerned with their own or their party’s public image.

*“If you appear with a [reimbursement] request after the election, the submission will not be considered to be such a big deal, so it will be delayed and so on. However, if you come during a season where the [election] campaign is ongoing, unfortunately, they see that they can benefit if they do something “good”, so there is more chance to get something. Which is something that’s really wrong.”[P10]*

In some cases, such political bravado is believed to have contributed to the set-up of separate patient hearings or the implementation of separate reimbursement processes for (ultra-)orphan drugs, in order to grant access for those that exceed the ICER threshold by allowing the inclusion of “softer” criteria to the assessment.

*“This is kind of a bit of a political game really and we have invented this [separate pathway] to allow us to meet the demands that the politicians are putting on us.”[P4]*

Participants also explained that often patients, their organizations and the wider public (such as parents of young rare disease patients) exert significant pressure on decision-makers through the media, in order to push for a positive reimbursement decision.

*“There was a controversial decision on the treatment for cystic fibrosis where the initial recommendation […] was not to approve it because it was just not cost-effective at all. But then the patient group lobbied with the government and the minister overturned that decision and made it available.” [P11]*

External pressure seems even more apparent when the decision impacts a mainly young patient population.

*“I would not say it happens a lot, but there have been some cases in history, Zolgensma* ^*®*^ *of course being one of them […] where it has kind of been insinuated to the committee that if a positive decision could be reached then it would be kind of good for public image.”[P8]*

Lastly, a market authorisation issued by the European Medicines Agency (EMA) may lead to implicit bias as it becomes even more difficult for decision-makers to deny reimbursement. Even though these decision-makers decide on (national) comparative cost-effectiveness or “value-for-money” rather than clinical effectiveness and safety, the public finds it difficult to accept that a decision-maker would “overrule” an international authority such as the EMA.

##### Perverse Effects

Apart from leading to implicit bias, a market authorisation granted by the EMA may also have a perverse impact by counter-intuitively demotivating further research, for instance into the cost-effectiveness of an orphan drug in a particular rare disease subgroup. Furthermore, it may discourage patients to participate in follow-up trials.

*“The patient, the family of these children say: “no, it was approved by the EMA, which means that it is something good for my child. So I do not accept that my child can be included in a clinical trial in which it can receive a placebo. I want Ataluren because it was approved, it was already assessed”. And so it is difficult because of the pressure of the parents, the society of the parents, which are funded by the industry of course. It is difficult to deny reimbursement.”[P7]*

In addition, ethical arguments in the context of the orphan drug legislation and status are often abused by companies that ask for high prices. This puts extra pressure on the decision-makers during the negotiation process.

*“We had only one orphan drug* *[…] there were almost no QALYs and it was very, very expensive […] but the company said “we can have a higher price because we are orphan”. [P18]*

As a result, several participants feel that orphan drug development has become a lucrative business model for the industry.

*“If you look at the business literature, and I am not talking about the academic literature but the glossy magazines, especially the American ones, each and every one of them has an article on how to make the most out of your orphan drug business model.” [P13]*

##### Ignoring the Opportunity Cost

Throughout the interviews it became clear that the complexity of orphan drug reimbursement may create a tunnel vision for decision-makers who wish to provide equal access to medicines for rare disease patients, simultaneously neglecting the large opportunity cost that may follow from reimbursing high-priced orphan drugs. When implementing separate pathways or other methods that create more flexibility for ultra-orphan drugs versus non-orphan drugs, decision-makers should be particularly careful not to, paradoxically, violate the equality principle.

*“What politicians and decision-makers don’t quite understand is the fact that if we have a limited amount of resources and when we start accepting more and more very expensive orphans and ultra-orphans, we are actually reallocating resources from a majority of patients whose treatments are relatively cheap, to a very small minority of patients whose treatments are very expensive. So there is the issue of equity and equality involved in this also.”[P13]*

There is a risk that, as a result of this tunnel vision, decision-making becomes opinion-rather than evidence-based, hereby unintentionally discriminating against non-rare disease patients who will ultimately carry the opportunity cost. For instance, participants warned against the anecdotal character of patient hearings as it adds extra subjectivity to the value assessment and ignores the opportunity cost.

*“The emotional approach should never prevail on the rational. Because doctors and regulators particularly should act as a pater familias […] [he] has to take care of all his children with equity. You cannot buy a Ferrari for a boy and leave the other on the bicycle.”[P7]*

#### Managed Entry Agreements as an External Factor Influencing Orphan Drug Appraisal

Apart from the contextual determinants, MEAs are another external factor that may influence the appraisal of orphan drugs. These agreements were often described as a welcome tool that have the potential to complement any VAF: by allowing a temporary reimbursement, by promising the collection of data on the orphan drug’s effectiveness and overall, by resulting in more reasonable prices and a more favorable budget impact. In general, they may allow a negative value assessment to lead to a positive appraisal, allowing the orphan drug to be reimbursed over a limited period of time, ideally followed by periodic reassessments of the orphan drug by analysing the collected data on cost and/or effectiveness. However, participants highlighted barriers that were similar to those mentioned before, such as a paucity of necessary data (to define realistic endpoints), a shortfall of expertise and negotiation power (as these agreements are complex to set up and monitor) and difficulty in defining specific criteria and setting up a clear framework (which would also allow for an efficient exit strategy if the orphan drug does not meet the conditions as agreed in the MEA). Moreover, due to the confidential character of the MEA, insufficient transparency on the reimbursement conditions and price results in a loss of oversight on the healthcare budget.

*“I once got a copy of a managed-entry agreement where at the very top it said managed-entry agreement. And then it was two pages of black. And then it was two signatures.”[P13]*

### Improving the Value Assessment and Appraisal of Orphan Drugs: Ways Forward as Proposed by Participants

Throughout the interviews, participants shared several conditions that decision-makers should take into account when aiming to improve any specific aspect of the reimbursement process for orphan drugs. For instance, when aiming to improve the inclusion of patients (aspect for improvement), the presence of an independent and well-developed patient organisation is assumed (preferred condition). Table 3 provides an overview of these preferred conditions linked to specific aspects of the orphan drug value assessment and appraisal framework.

**TABLE 3:** Aspects of the reimbursement (value assessment and appraisal) process with room for improvement and the preferred conditions linked to each improvement, as voiced by interviewees.

Practice or aspect for improvement of the reimbursement framework	Preferred conditions
Involvement of multiple stakeholders	Only if involvement of each stakeholder category is subjected to strict criteria regarding preparation and conflict of interest
Including patients	Only if there is an independent and well-organized patient organisation
	Only when also including a “counter-voice”: an independent representative of the public who defends the opportunity cost
Balancing the role of the state	Only when the decision-making committee consists of a sufficient and representative sample of stakeholders
	Only when all stakeholders are sufficiently prepared
Improving negotiation power	Only if there is a clear reimbursement framework
	Only if there are enough human resources
	Only if these human resources have sufficient expertise
International cooperation for price negotiations	Only when EU legislation is appropriately adapted and if countries agree to one price
Improving transparency	Only when the overall reimbursement framework, its criteria and their potential weights are clear
	Only if the pharmaceutical company’s right on proprietary information is guaranteed during price negotiations
Publication of the report	Only if there are enough human resources to prepare the report
	Only if the report also includes the discussion that took place during the appraisal
	Only if the report is adapted for a various and variable audience: (i) a version containing the full report, (ii) a version adapted for clinical experts, (iii) a version intended for a lay person or a patient
Improving framework structure	Only fix criteria and weights if there is a broad consensus
	Only allow more flexibility for ultra-orphan drugs when there is empirical
Implementing multi-criteria decision-analysis	Only if the framework is set-up in a pragmatic manner, with room for flexibility and continous improvement
Implementing cost-effectivity analysis	Only if there are enough human resources to perform the analysis
	Only if there is enough expertise to perform the analysis
Weighing QALYs or varying the ICER threshold	Only if utilities are available
	Only if weights and thresholds are empirically defined
	Only if there is consensus on the arguments in favor of weighing or varying
	Only if arguments in favor of weighing or varying are fully and transparently communicated
	Only if the budget is not fixed
Accepting a higher ICER threshold for (ultra-)orphan drugs	Only if overall budget impact is lowered by means of a MEA
Concluding MEAs	Only if outcomes are adequately monitored
	Only if there is a clear exit strategy
Setting up an MEA according to a risk-sharing model	Only if there are clear agreements on endpoints, stop-criteria and consequences
Setting up and maintaining registries	Only if extra workload for stakeholders such as clinicians is avoided, for instance by entering data only once

Abbreviations: ICER, incremental cost-effectiveness threshold; MEA, managed entry agreement; QALY, quality-adjusted life year

In addition, Table 4 presents an extensive number of recommendations for the improvement of the value assessment and appraisal process of orphan drugs, which were shared by the participants.

**TABLE 4:** Recommendations for optimizing the reimbursement process as shared by experts on orphan drug reimbursement.

**Improve the availability of data**
Centralize data in order to improve its availability
Streamline access to aggregated data, in order to improve accessibility
Use aggregated health data to focus on orphan drugs that provide added value
Focusing on the benefits by means of the QALY over the ICER
Enable international collaboration for the validation of EQ-5D or for defining QALYs
**Support the development of national expertise**
Provide better training on pharmaco-economic principles, for instance on-the-job or even by organising a dedicated master’s degree at local universities
**Optimize the structure of the value assessment framework**
Define clear reimbursement criteria
If quantification, categorisation or weighing of criteria is desired, set up a population wide survey or special multi-stakeholder task force in order to obtain consensus
Focus on the improvement of the process, through increased structure and transparency, rather than on optimizing existing methods such as the cost-effectiveness analysis
Allow a certain degree of flexibility to enable continuous improvement of the framework
Adopt a critical attitude rather than strictly adhering to criteria and benchmarks
Opt for a deliberative process through a decision-tree over a rigid MCDA framework
During each reimbursement decision, draw parallells with previous decisions on similar cases in order to improve consistency in decision-making
**Increase transparency**
Communicate transparently on reimbursement criteria
Include all arguments raised during the appraisal, either against or in favor of reimbursement, in the HTA report
Communicate openly about uncertainties
Learn to say no when evidence is not sufficient
**Minimize risk of bias**
Formalize the inclusion of patients
Implement methods to make patient-based evidence more robust
Include a wider patient group, consisting of patient representatives with and without the disease
Involve an ethical expert or committee overseeing the patient involvement, or if feasible, the entire reimbursement process
Exclude stakeholders from specific parts of the assessment whenever a conflict of interest is identified
Record any attempt of internal or external stakeholders to illicitly influence the reimbursement decision, either by registration an internal document or as part of the HTA or reimbursement report
**Considering the opportunity cost**
Adopt a principle of fairness behind the treatment of orphan vs. non-orphan drugs, treating both drug categories equally
If patient hearings are organised, do so equally for both categories
Request pharmaceutical companies to dedicate a part of the submission to the opportunity cost of the orphan drug under evaluation
**Balance responsibilities**
Allow universities to support HTA
Implement strict criteria for multiple stakeholders partaking in decision-making regarding dedication of time for preparation
**Improve collaboration between stakeholders**
Stimulate further development of international joint negotiation partnerships such as the Beneluxa Initiative or FINOSE
Share workload on the evaluation of clinical aspects of the HTA
Share best practices between HTA and reimbursement agencies
Streamline the EMA and HTA processes, enable collaboration at an early development stage
Improve communication between authorities and the industry by allowing in-process consultation
Regularly ask the pharmaceutical industry for feedback on the reimbursement framework and, in particular, on communication methods
**Balancing negotiation power**
Improve international collaboration between HTA agencies (see previous)
Define an international maximum price for specific orphan drugs
Clearly define national reimbursement criteria (and weights, were applicable)
**Managed entry agreements**
Set specific targets
Provide adequate IT infrastructure for monitoring
Provide (human and financial) resources for monitoring
Implement pay-for-performance schemes
Set clear guidelines for discontinuation of treatment when the orphan drug does not reach the specified targets at the end of the contract term

Abbreviations: EMA, European Medicines Agency; EQ-5D, EuroQol 5D; HTA, health technology assessment; ICER, incremental cost-effectiveness threshold; MCDA, multi-criteria decision analysis; QALY, quality-adjusted life year.

**FIGURE 1:**
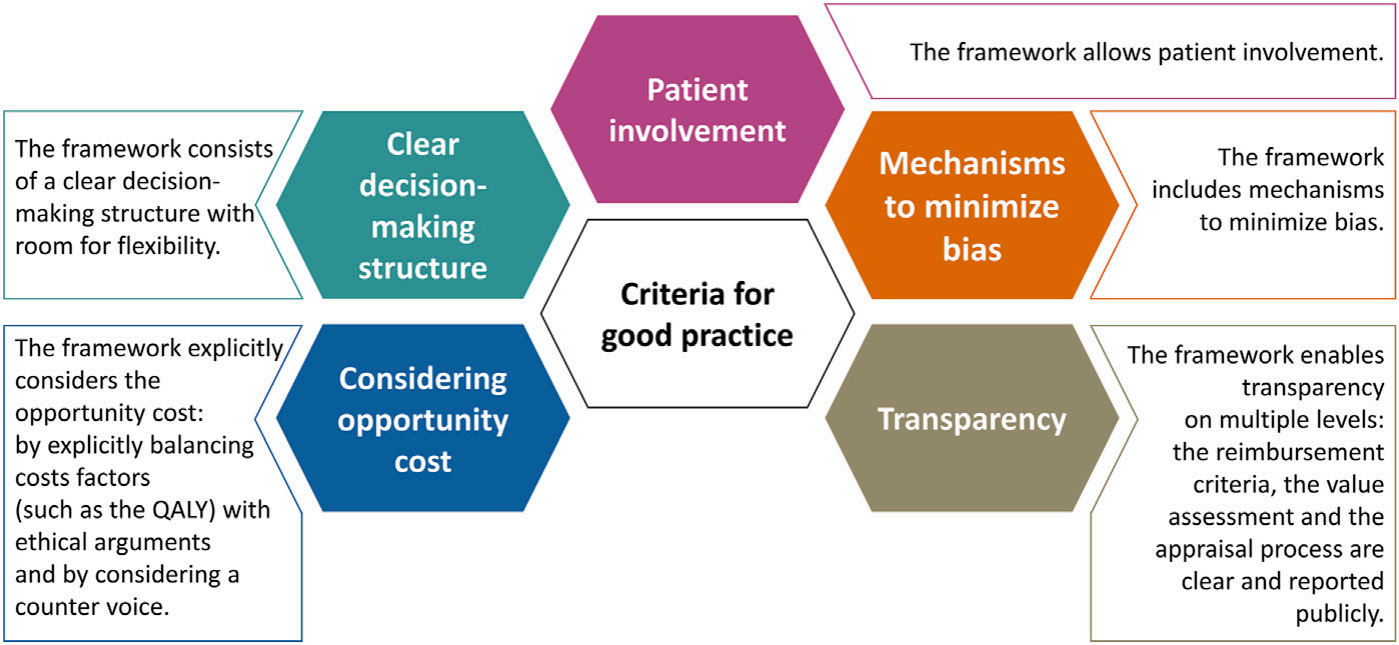
Five criteria that may signify a good practice when setting up a reimbursement (value assessment and appraisal) process for orphan drugs.

Finally, Figure 1 presents five criteria that may be considered to signify a “good practice” when setting up a framework for the value assessment and appraisal of orphan drugs. These criteria for good practice are non-exhaustive and were derived directly from the data analysis. Table 5 shows the performance of the participating jurisdictions or countries against these criteria on an aggregated level. For each good practice, the colored indicator shows whether these were predominantly present (green), whether the current practice could be improved (orange) or whether the good practice was overall absent (red). Unfortunately, the lack of a green indicator suggests that none of the criteria were present throughout the majority of countries included.

**TABLE 5:** The overall performance of 19 European jurisdictions against five criteria of good practice related to the reimbursement process of orphan drugs.

Criteria for good practice	Indicator	Rationale
1. Transparency on multiple levels	orange	The reimbursement criteria are publicly available in the majority of the jurisdictions. In addition, although a report is published in most of the jurisdictions, many of them include only a summary of the decision. The discussion that took place during the appraisal is usually not included
2. Patient involvement	orange	Participants from half of the jurisdictions indicated that they currently involve patients either through the appraisal committee or by organising patient hearings. In others, patients are either not involved or heard occasionally through communication with the health insurance fund or doctors. There were no indications that evidence contributed by patients was standardized in any of the jurisdictions
3. Clear decision-making structure with room for flexibility	orange	Nearly half of the jurisdictions lack a clear decision-making framework. In the others, several have implemented a checklist and a minority adopted a score-card method where criteria are weighed through scoring. It is not clear to what extent these frameworks allow room for continuous improvement
4. Mechanisms to minimize bias	red	Overall, countries have few mechanisms in place to ensure that bias is minimized. One participant shared “good” practices such as discussing and registering internally whether committee members perceived external attempts to exert pressure on the decision. Another “good” practice is the exclusion of stakeholders from a part of the process in the case of a conflict of interest
5. Explicitly considering opportunity cost	red	Overall ill considered

## Discussion

Whereas previous research in this area has focused on the reimbursement frameworks for orphan drugs in general – their identification, description and legitimacy – (Nicod et al., 2020a; Zelei et al., 2016; Bourke et al., 2018; Picavet et al., 2014b; Szegedi et al., 2018; Nicod et al., 2019; Hughes et al., 2005; Hughes et al., 2007; Wagner et al., 2016; Annemans et al., 2017; Drummond and Towse 2014; Schey et al., 2017; Simoens 2012; Simoens et al., 2012; Degtiar 2017; Rosenberg-Yunger et al., 2011; Denis et al., 2010; Albertsen 2021; Schey et al., 2020) the present study investigated how experts perceive the reimbursement (value assessment and appraisal) process for orphan drugs in their country and how it can be optimised. In this regard, it has identified the positive attributes, barriers and external factors that impact the process. Whereas a recent theoretical study identified the strengths and weaknesses related to specific VAFs for orphan drugs (Blonda et al., 2021), the main strength of the present study lies in the primary nature of the data, which were generated directly from the experiences and viewpoints from experts on orphan drug reimbursement, throughout 19 different countries. By including an expert group consisting of researchers, professors and health economists, amongst others, the study provides a bottom-up approach towards improving the reimbursement of orphan drugs.

### Positive Attributes and Barriers Related to the Reimbursement Process of Orphan Drugs

Overall, the main positive attributes, as summarized in Table 2, were in line with findings as described in several publications on the access of orphan drugs (Simoens 2011; Godman et al., 2018; Pearson et al., 2018; Schuller et al., 2015; Mincarone et al., 2017; Nicod et al., 2017; Blonda et al., 2021).

For instance, participants generally believed that ethical considerations such as disease severity and unmet need should play a significant role when considering an orphan drug’s reimbursement. However, consistent with existing literature, the trade-off between ethical and economic considerations remains debatable (McCabe 2010; Henderson et al., 2020; Picavet et al., 2013; Albertsen 2021; McCabe et al., 2005; Drummond and Towse 2014). In addition, our results remained inconclusive as to whether disease rarity legitimizes the implementation of a separate framework for (ultra-)orphan drugs or to what extent this criterion warrants more flexibility when assessing these treatments. As a way forward, the implementation of decision-making frameworks such as multi-criteria decision analysis would allow a fair and consistent evaluation of these arguments (Blonda et al., 2021; Zimmermann et al., 2021).

Furthermore, while participants valued the involvement of patients, as a means to balance the remaining uncertainties in the evidence base, many struggled with the specifics of such involvement, especially regarding when, which and how patients should be involved in the HTA. This hesitation is also reflected in the overall performance of the jurisdictions regarding patient involvement as a “good practice” to orphan drug reimbursement (Table 5). As Drummond et al. described, the current, overall approach towards patient involvement is considered to be “reactive rather than proactive” (Douglas et al., 2015; Drummond et al., 2020). Furthermore, the choice for a specific set of patients (those with or without the diseases) may be culturally defined (Torbica et al., 2017; Drummond et al., 2020). Although there has been increasing guidance on patient involvement, as well as interest in tools to make overall patient involvement more robust, for example via patient-reported “outcome” or “experience” measures (PROMs and PREMs), their use in value assessment/HTA is still limited (Hunter et al., 2018; Janssens et al., 2019; Drummond et al., 2020; Jommi et al., 2021; Wale et al., 2021).

Also, the barriers towards orphan drug reimbursement have been described extensively in literature (Drummond et al., 2007; Simoens 2011; Sussex et al., 2013b; Iskrov et al., 2017). However, the present study provided new insights into the importance of cultivating trust between stakeholders in order to maximise data sharing, as a means to expand the evidence base which is often scarce in orphan drugs - and ultra-orphan drugs especially. Overall, decision-makers should keep in mind that if they do not anticipate on the identified barriers, stakeholders may ultimately question not only the cost-effectiveness of orphan drugs, yet the cost-effectiveness of the overall reimbursement process.

### The Price of Orphan Drugs

Consistent with literature, participants expressed their concern over the high price of orphan drugs and the secrecy surrounding the real cost of developing an orphan drug (Roos et al., 2010; Hughes-Wilson et al., 2012; Michel and Toumi 2012; Picavet et al., 2014a; Luzzatto et al., 2018; Brenna et al., 2020). However, in recent years an increasing amount of effort has been spent to gain more insight into the real price of drugs (DiMasi et al., 2016; Prasad and Mailankody 2017; Wouters et al., 2020) and orphan drugs (Jayasundara et al., 2019; Schlander et al., 2021). Still, the lack of transparency on the agreed price under a MEA creates information asymmetry between the payer and the pharmaceutical company, which in turn further reduces the negotiation power of the payer (Vogler and Paterson 2017; Ahmad et al., 2020). In addition, we hypothesize that the lack of transparency on the real price of an orphan drug decreases accountability on whether it is worth its investment, in particular given the uncertainty in effectiveness. Therefore we advise payers to increase the transparency on the overall reimbursement process by considering the recommendations as provided in Tables 3 and 4. In addition, we believe that individual payers may increase their negotiation power - in order to set a fair price - through international cooperation, for instance by sharing the actual paid prices through a formal EU drug pricing transparency initiative (Vogler and Paterson 2017; Ahmad et al., 2020; Jommi et al., 2021; Russo et al., 2021). However, they should be careful to include the pharmaceutical industry as an equal partner when setting up such initiatives, in order to avoid unwanted effects on the availability or affordability of orphan drugs (Ahmad et al., 2020; Riccaboni et al., 2020).

### The Impact of Contextual Determinants on the Appraisal of Orphan Drugs

A recent publication on the reimbursement of nusinersen identified the presence of contextual determinants in the fact that the orphan drug was reimbursed in all jurisdictions under study, despite unfavourable value assessment (Blonda et al., 2022). Our results provided an in-depth exploration of these contextual determinants - bias, perverse effects and disregarding the opportunity cost - that act as confounding factors that may influence the positive attributes, barriers and other external factors (such as the MEAs), as well as the reimbursement process as a whole. As a result, they often have the power to deviate a negative or uncertain value assessment into a positive appraisal. The role of these contextual determinants in creating this discrepancy and the extent to which they impact the appraisal often remain unclear to stakeholders and the wider public, as these determinants are implicit by nature. Hence, they decrease transparency and contribute to unaccountable decision-making, which may result in disputes and public outcry. Moreover, it may lead to insecurity among stakeholders and the wider public concerning the motivation behind the final decision, which becomes dependent on a case-by-case assessment and may be subjected to cultural, historical and political whims. As a solution, we advise decision-makers to adopt an MCDA framework during the appraisal process, as this allows all arguments against/in favour of reimbursement to be captured explicitly and to be referred to afterwards, in case of doubt.

### Managed Entry Agreements

Although remaining uncertainties concerning (cost-) effectiveness may impact orphan drugs more significantly compared to non-orphan drugs at time of assessment, we believe this to be an inaccurate argument for decision-makers to create an adjusted or separate reimbursement pathway for orphan drugs (Blonda et al., 2021). Rather, we argue that decision-makers may manage such uncertainties through the use of MEAs. Unfortunately, the present study confirms that MEAs are often applied as a tool for cost-containment (for instance by means of a simple price discount scheme). Although this may temporarily give decision-makers some security on the financial burden of reimbursing an orphan drug, it will not solve the main uncertainties on its cost-effectiveness, as no real-world evidence is being collected and reassessed. Moreover, decision-makers have no objective grounds for disinvestment of an orphan drug if it fails to meet the patients’ needs. By focusing only on cost-containment, the potential of MEAs is reduced to being an *ad-hoc* solution: a (temporary) band-aid that attempts to cover the knowledge gap that may emerge during the drug appraisal process. Rather, we advise decision-makers to allow MEAs to become an integral part of the reimbursement process, by focusing on outcome-/performance-based (risk-sharing) schemes, or a scheme that combines performance-with financial-based reimbursement conditions. We further urge decision-makers to implement these schemes setting out a long-term vision, demanding a periodic re-evaluation of the orphan drugs under contract and providing transparency in the outcomes of the reassessment (Jommi et al., 2021), while considering the recommendations as provided in this study.

### Improving the Reimbursement Process of Orphan Drugs

Many of the strengths and barriers are connected to the current state of the art on the methods that shape the reimbursement process and its theoretical implementation. As such, improvements on this level are time and resource intensive and, as the results indicated, subject to progressive insight and hence, continuous improvement. Meanwhile, contextual determinants such as bias, perverse effects or the consideration of opportunity cost are more universal by nature and thus, it may be easier to limit their impact by making small adjustments. Herein lies the key to improving the efficiency of the reimbursement process: by decreasing the impact of the contextual determinants on the appraisal rather than choosing new methods to improve the theoretical value assessment/HTA process. For example, rather than debating on methods to weigh QALYs according to specific ethical arguments such as severity or unmet need, decision-makers could provide more transparency on which ethical arguments where taken into consideration during decision-making, and why.

Our empirical study shows that, given the high costs and uncertainties related to the reimbursement of orphan drugs, participants generally expect decision-makers to implement a reimbursement framework that provides sufficient transparency and accountability in order to ascertain the legitimacy of the reimbursement decision. For this reason, previous studies have highlighted the importance of aligning the reimbursement process of orphan drugs to the principles of “Accountability for Reasonableness” (A4R) (Daniels 2001; Wagner et al., 2019; Blonda et al., 2021). In this context, we believe that overall, our results are in alignment with, and therefore confirmed by the A4R framework as it is based on the following four principles: 1) transparency on all levels of the reimbursement process; 2) the flexibility that allows the continuous improvement of the framework according to new insights; 3) the inclusion of criteria and arguments (in favour or against reimbursement) that are relevant, reasonable, based on reliable data and, thus, supported by the stakeholders involved in the process; and 4) a proper execution and follow-up (for instance of the conditions as agreed upon in the MEA) of the framework through appropriate policy measures, regulation and enforcement (Blonda et al., 2021). In addition, the present study provides a distinctive contribution to the state-of-the-art since it provides concrete steps for decision-makers to align their reimbursement process to the principles of the A4R framework. As they were generated bottom-up, they will increase the accountability and hence, legitimacy of the appraisal, hereby improving the reimbursement process as a whole.

### Limitations

Our study is not without limitations. First of all, while we included an even, geographical spread of jurisdictions, we were not able to include participants from several major European countries such as France, Germany and the Netherlands despite sustained efforts to do so. Second, a language barrier may have limited some participants to nuance their viewpoints and recommendations during the interviews. However, during the iterative consultation rounds, we aimed to identify and discuss these points in order to find more nuance. Nevertheless, the language barrier may have resulted in stronger conclusions on some aspects of the concepts as discussed in the results.

## Conclusion

Through participant experience, this study has underlined the importance of transparency and trust during the reimbursement of orphan drugs. Furthermore, it highlighted the need for a clear decision-making framework while allowing room for continuous improvement. Moreover, it identified several contextual determinants that impact the appraisal process in particular, such as the influence of bias and an overall ill consideration of the opportunity cost (of orphan drug versus non- orphan drugs). Whereas the identified positive attributes and barriers mainly relate to the methods that guide the orphan drug assessment, the appraisal process and its structure, the contextual determinants are more universal by nature, as they are not related to the reimbursement process directly. Hence, we advise decision-makers to focus on limiting the impact of these contextual determinants and to adhere to the principles of A4R when aiming to improve their reimbursement process, by making use of the extensive recommendations as provided in this publication.

## Data Availability

The datasets presented in this article are not readily available because the data collected during this preference study are stored in the secured database Sharepoint at KU Leuven. The study adheres to national data protection laws and the identity of the participants is kept strictly confidential. Coded data can only be shared with the researchers involved in the study. These provisions are defined in the research protocol, which was approved by the Ethical Committee (EC) Research University Hospitals Leuven (6/05/2020, S63958). Any requests concerning the coded datasets should be directed to alessandra.blonda@kuleuven.be.
